# Time to Sustained Improvement in Bowel Movement Frequency with Telotristat Ethyl: Analyses of Phase III Studies in Carcinoid Syndrome

**DOI:** 10.1007/s12029-020-00375-2

**Published:** 2020-03-07

**Authors:** Joseph S. Dillon, Matthew H. Kulke, Dieter Hörsch, Lowell B. Anthony, Richard R. P. Warner, Emily Bergsland, Staffan Welin, Thomas M. O’Dorisio, Pamela L. Kunz, Chad McKee, Pablo Lapuerta, Phillip Banks, Marianne Pavel

**Affiliations:** 1 Department of Internal Medicine – Endocrinology and Metabolism, University of Iowa, Iowa City, IA USA; 2 Division of Medical Oncology, Boston Medical Center, Boston, MA USA; 3 Department of Gastroenterology/Endocrinology, Center for Neuroendocrine Tumors, Zentralklinik Bad Berka, Bad Berka, Germany; 4 Division of Medical Oncology, University of Kentucky, Lexington, KY USA; 5 Division of Gastroenterology, Icahn School of Medicine at Mount Sinai, New York, NY USA; 6 Department of Medicine, University of California, San Francisco, San Francisco, CA USA; 7 Department of Endocrine Oncology, Uppsala University Hospital, Uppsala, Sweden; 8 Department of Medicine – Division of Oncology, Stanford University, Palo Alto, CA USA; 9 Lexicon Pharmaceuticals, Inc., The Woodlands, TX USA; 10 Department of Hepatology and Gastroenterology, Charité–Universitätsmedizin, Berlin, Germany; 11 Department of Medicine 1, Division of Endocrinology, Friedrich-Alexander-Universität, Erlangen-Nürnberg, Erlangen, Germany

**Keywords:** Malignant carcinoid syndrome, Tryptophan hydroxylase, Telotristat ethyl, Neuroendocrine tumors, Diarrhea, Serotonin

## Abstract

**Background:**

Telotristat ethyl is approved to treat carcinoid syndrome diarrhea in combination with somatostatin analogs. In TELESTAR and TELECAST phase III studies, patients with carcinoid syndrome received telotristat ethyl 250 or 500 mg 3 times per day (tid) or placebo tid in addition to somatostatin analogs. The aim of this prespecified analysis was to examine the time to reductions in bowel movements (BMs) in the TELESTAR and TELECAST studies using survival analysis methods.

**Methods:**

First occurrence of sustained response was defined as the time to the first day of 2 consecutive weeks with a mean BM frequency improvement of ≥ 30% from baseline during the 12-week double-blind treatment periods. Time to first ≥ 30% worsening in BM frequency was also measured. Treatments were compared with the log-rank test; Cox regression models provided point and confidence interval estimates of the hazard ratios for each trial.

**Results:**

In TELESTAR and TELECAST, majority of patients (69%) on telotristat ethyl experienced a sustained ≥ 30% improvement in BM frequency. The median time to sustained reduction of at least 30% in BM frequency was significantly faster (fewer days to onset) for telotristat ethyl compared with placebo in both TELESTAR (250 mg, HR = 2.3 [95% CI, 1.3–4.1, *P* = 0.004]; 500 mg, HR = 2.2 [95% CI, 1.2–3.9, *P* = 0.009]) and TELECAST (250 mg, HR = 3.9 [95% CI, 1.6–11.1, *P* = 0.003]; 500 mg, HR = 4.2 [95% CI, 1.7–11.7, *P* = 0.002]). In TELECAST, 42% of patients on placebo experienced sustained worsening in BM frequency compared with 20% on telotristat ethyl; no significant difference was observed in TELESTAR.

**Conclusion:**

The time of onset of sustained BM frequency improvement mean and range are important when considering use of telotristat ethyl in patients with carcinoid syndrome diarrhea. Telotristat ethyl may also reduce sustained worsening in BM frequency.

**Trial Registration:**

ClinicalTrials.gov Identifiers: NCT01677910, NCT02063659

## Introduction

Patients with symptomatic carcinoid syndrome diarrhea can experience a high frequency of bowel movements (BMs), with one study reporting 5.0 ± 2.7 diarrhea episodes per day in 40 patients, prior to their initiation of somatostatin analog (SSA) therapy [1]. Diarrhea seriously impacts quality of life for these patients; 30% of patients will continue to have debilitating symptoms despite initiation of SSA therapy, while more will lose responsiveness to SSA therapy at indicated doses [2–4].

Telotristat ethyl is an oral tryptophan hydroxylase inhibitor recently approved by the United States (US) Food and Drug Administration (FDA) and European Medicines Agency/European Commission for the treatment of carcinoid syndrome diarrhea in combination with SSA therapy in adults whose symptoms are inadequately controlled by SSA therapy [5, 6]. It has also been added to the US National Comprehensive Cancer Network (NCCN) guidelines for treatment of carcinoid syndrome-related diarrhea [7]. Its efficacy xand safety were evaluated in two phase III studies, TELESTAR and TELECAST, in patients with carcinoid syndrome. Significant reductions in BM frequency were demonstrated for patients taking telotristat ethyl in both studies [8, 9].

The pivotal, randomized phase III trial (TELESTAR) that resulted in FDA approval determined the efficacy of telotristat ethyl in reducing daily BM frequency from baseline [5, 8]. Responses, predefined as a BM frequency reduction of ≥ 30% from baseline for ≥ 50% of the double-blind treatment (DBT) period, were observed in 44% and 42% of patients who received telotristat ethyl 250 mg three times per day (tid) or telotristat ethyl 500 mg tid, respectively, compared with only 20% of patients who received placebo [8].

SSAs are considered first-line therapy to treat neuroendocrine tumors (NETs) and carcinoid syndrome [10, 11]. The majority of study patients receiving telotristat ethyl in the two trials were also on stable doses of SSAs as standard of care [8, 9]. SSAs improve carcinoid syndrome symptoms by interacting with extracellular receptors on NET cells, thereby regulating serotonin and other hormone release [10, 11]. Telotristat ethyl improves carcinoid syndrome diarrhea by intracellular inhibition of tryptophan hydroxylase and blocks serotonin production within NET cells [12]. Although the medications are potentially complementary in treating carcinoid syndrome-related diarrhea, the underlying mechanisms differ, and the drugs may have different time courses of effect. Inaccurate expectations by patients and health care providers regarding the rapidity of onset of the effects of telotristat ethyl could lead to either inappropriate early discontinuation or prolonged use of medication. Thus, it is important to measure the onset of effect and potential for sustained improvement.

Although the main safety and efficacy results of telotristat ethyl use have already been published, the timing of responses has not been addressed in detail [8, 9]. Therefore, the objective of this prespecified analysis was to examine the time to achievement of BM frequency reductions in the TELESTAR and TELECAST studies using survival analysis methods. Median times to achievement of effect on related gastrointestinal symptoms, including changes in urgency and stool form, were also estimated.

## Methods

The designs of the TELESTAR and TELECAST studies, which differ only in inclusion criteria, have been detailed elsewhere [8, 9]. Briefly, patients with a history of well-differentiated NETs were eligible for TELESTAR if they had a mean of ≥ 4 BMs per day while on stable-dose SSA therapy. Patients were eligible for TELECAST, which was intended for those not qualifying for TELESTAR, if they had < 4 BMs per day during the screening period and were receiving SSA therapy. Additionally, in the TELECAST study, patients with ≥ 4 BMs per day were eligible if they were not receiving SSA therapy. All patients in TELECAST had carcinoid syndrome that required additional treatment, primarily owing to gastrointestinal symptoms.

In both studies, baseline BM frequency was assessed with electronic diaries over a 3- to 4-week screening period. Qualifying patients were then randomized to placebo tid, telotristat ethyl 250 mg tid, or telotristat ethyl 500 mg tid (while continuing stable-dose SSA therapy, if already receiving SSA). Therapy was administered in a double-blinded fashion for 12 weeks. In the 500-mg tid dosage arm, there was blinded titration, with patients receiving 250 mg tid during the first week. After the completion of the 12-week DBT period, all patients were offered open-label telotristat ethyl 500 mg tid.

The primary endpoint in the TELESTAR study was the change from baseline in BM frequency, averaged over 12 weeks. The TELECAST study had co-primary endpoints of safety and changes in urinary 5-hydroxyindoleacetic acid (5-HIAA). Changes in BM frequency were a secondary endpoint in TELECAST and were analyzed in the same way as in TELESTAR.

The development of Kaplan–Meier curves for sustained reduction in BM frequency was prespecified in both studies. Sustained reduction was defined as the time from the first double-blind dose to the first day of two consecutive weeks (i.e., 14 days) with BM frequency of at least 30% below the individual baseline mean.

The 30% threshold was selected based on prior literature on BM frequency and patient-reported outcomes in patients with NETs [2]. Further support of this threshold came from patient interviews conducted among participants in a phase II study of telotristat ethyl and from an expert panel of physicians reviewing these data and their own experience [13]. The log-rank test was applied for both studies to compare the time to reduction in BM frequency among treatment arms.

Prespecified analyses also addressed the time to sustained worsening of BM frequency, using the same 30% threshold. This was prespecified for the TELESTAR and TELECAST studies because in a phase II study of telotristat ethyl, there was the suggestion of worsening of BM frequency observed on placebo [14].

In the TELESTAR and TELECAST studies, urgency to defecate was recorded daily on electronic diaries through a yes/no question. Stool form was also measured daily with the Bristol Stool Form 7-point scale [15, 16]. Mean changes in stool form have been previously presented for TELESTAR and TELECAST [8, 9], whereas mean changes in urgency to defecate have been reported for TELESTAR and collected for TELECAST [8, 17]. Differences between treatment groups were examined using least squares means. Differences were also examined at each week among patients who achieved ≥ 30% reductions in BM frequency, those who achieved 0–30% reductions in BM frequency, and those who had worsened BM frequency.

No adjustment for multiplicity was performed, as these analyses were intended to further qualify changes in BM frequency, which was already a primary endpoint in TELESTAR and a secondary endpoint in TELECAST.

## Results

Baseline characteristics of patients in the TELESTAR and TELECAST studies are presented in Table 1. The key difference between the studies was the inclusion criteria for baseline BM frequency. In TELESTAR, the overall mean (SD) baseline BM frequency was 5.7 (1.8) BMs per day (range 3.5–11.5) and in TELECAST, it was 2.5 BMs per day (range 0.88–5.53). A total of 211 patients were treated in these 2 trials: 71 patients were randomized to receive placebo, 70 to telotristat ethyl 250 mg tid, and 70 to telotristat ethyl 500 mg tid; all patients were on SSA therapy, except for three patients in the telotristat ethyl 250 mg tid arm and five patients in the telotristat ethyl 500 mg tid arm in the TELECAST study [8, 9].

**Table 1:** Baseline characteristics

		TELESTAR			TELECAST		
Characteristic	Statistic	Placebo (*N* = 45)	Telotristat ethyl 250 mg (*N* = 45)	Telotristat ethyl 500 mg (*N* = 45)	Placebo (*N* = 26)	Telotristat ethyl 250 mg (*N* = 25)	Telotristat ethyl 500 mg (*N* = 25)
u5-HIAA (mg per 24 h)	*n*	44	42	44	26	25	25
	Mean (SD)	80.97 (161.01)	92.65 (114.90)	89.50 (144.47)	81.99 (113.61)	86.30 (73.51)	66.05 (88.94)
BMs (counts per day)	*n*	45	45	45	25	25	25
	Mean (SD)	5.20 (1.35)	6.09 (2.07)	5.81 (1.96)	2.19 (0.67)	2.53 (1.25)	2.79 (1.57)
Stool form score^a^	*n*	45	45	45	25	25	25
	Mean (SD)	5.92 (0.70)	5.93 (0.50)	5.97 (0.69)	4.97 (0.91)	5.11 (0.84)	5.29 (0.83)
Urgency/immediate need to defecate (proportion of days)	*n*	38	39	38	26	25	25
	Mean (SD)	0.88 (0.16)	0.84 (0.24)	0.85 (0.27)	0.40 (0.37)	0.29 (0.28)	0.44 (0.37)

Abbreviations: *BM*, bowel movement; *SD*, standard deviation; *u5-HIAA*, urinary 5-hydroxyindoleacetic acid

^a^Bristol Stool Form Scale [16]

Significant BM frequency reductions with telotristat ethyl compared with placebo were seen in both studies, as were significant reductions in BM frequency from baseline [8, 9]. At week 12 in the TELESTAR study, the mean (SD) BM frequency changes from baseline were − 0.9 (1.2), − 1.7 (1.7), and − 2.1 (1.9) BMs per day on placebo (SSA alone), telotristat ethyl 250 mg tid, and telotristat ethyl 500 mg tid, respectively (*p* < 0.001 for each compared with baseline) [8]. At week 12 in the TELECAST study, the mean (SD) BM frequency changes from baseline were + 0.06 (0.5), − 0.79 (1.0), and − 0.85 (1.0) BMs per day on placebo (*p* = 0.44, compared with baseline), telotristat ethyl 250 mg tid (*p* = 0.008, compared with baseline), and telotristat ethyl 500 mg tid (*p* < 0.001, compared with baseline), respectively [9].

**Fig. 1:**
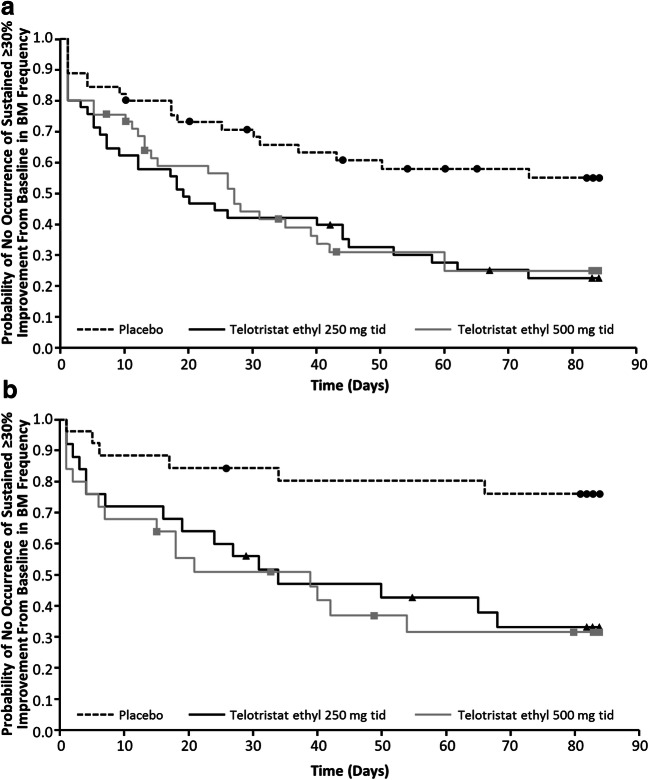
Kaplan–Meier curves for improvement in BM frequency. Probability with time that a patient would not have a sustained ≥ 30% improvement in BM frequency for patients in the TELESTAR (**a**) and TELECAST (**b**) studies. A sustained ≥ 30% improvement from baseline was defined as the time from the first DBT dose date to the first day of a consecutive 14-day period with BM frequency at least 30% below the individual baseline BM frequency. Patients having no ≥ 30% improvement were censored on the last day of the last week a valid assessment of the endpoint can be made. Shapes indicate censored values. Abbreviations: BM, bowel movement; DBT, double-blind treatment; tid, three times per day

The median time to sustained reduction of at least 30% in BM frequency was significantly faster (fewer days to onset) for telotristat ethyl compared with placebo in both TELESTAR (Fig. 1a and Table 2) and TELECAST (Fig. 1b and Table 2). In TELESTAR, the median times to first occurrence of sustained ≥ 30% improvement were 19 days and 27 days for the telotristat ethyl 250 mg tid (HR = 2.3, 95% CI, 1.3–4.1, *p* = 0.004) and telotristat ethyl 500 mg tid treatment groups (HR = 2.2, 95% CI, 1.2–3.9, *p* = 0.009), respectively (*n* = 45 for both groups). In TELECAST, the median times to improvement were 34 days and 39 days for the telotristat ethyl 250 mg tid (HR = 3.9, 95% CI, 1.6–11.0, *p* = 0.003) and telotristat ethyl 500 mg tid groups (HR = 4.2, 95% CI, 1.7–11.7, *p* = 0.002), respectively (*n* = 25 for both groups). Median time to sustained improvement was not reached on placebo in either study.

**Table 2:** Number of days to first occurrence of a sustained ≥ 30% improvement from baseline in BM frequency during the DBT period

	TELESTAR			TELECAST		
Statistic	Placebo (*n* = 45)	Telotristat ethyl 250 mg (*n* = 45)	Telotristat ethyl 500 mg (*n* = 45)	Placebo (*n* = 26)	Telotristat ethyl 250 mg (*n* = 25)	Telotristat ethyl 500 mg (*n* = 25)
Patient with an event, *n*	19	34	31	6	16	16
Censored, *n*	26	11	14	20	9	9
25th percentile (95% CL)^a^, days	18.0 (4.0, 50.0)	5.0 (1.0, 12.0)	10.0 (1.0, 15.0)	NR	7.0 (3.0, 31.0)	6.0 (1.0, 21.0)
Median percentile (95% CL)^a^, days	NR	19.0 (9.0, 44.0)	27.0 (13.0, 40.0)	NR	34.0 (NC)	39.0 (NC)
75th percentile (95% CL)^a^, days	NR	73.0 (NC)	NR	NR	NR	NR
HR (95%), telotristat ethyl vs placebo^b^	–	2.3 (1.3, 4.1)	2.2 (1.2, 3.9)	–	3.9 (1.6, 11.0)	4.2 (1.7, 11.7)
*p* value^c^	–	0.004	0.009	–	0.003	0.002

Abbreviations: *BM*, bowel movement; *CL*, confidence limits; *DBT*, double-blind treatment; *HR*, hazard ratio; *NC*, not calculated; *NR*, not reached; *u5-HIAA*, urinary 5-hydroxyindoleacetic acid

^a^Median, percentiles, and 95% CL are from Kaplan–Meier estimates.

^b^HR and 95% CL are from Cox proportional hazards regression model, which includes treatment group and u5-HIAA stratification at randomization as fixed effects.

^c^p -value is from log-rank test stratified by u5-HIAA at randomization.

The timing of response varied widely among patients. In TELESTAR, the earliest sustained improvement in BM frequency was observed at three days of telotristat ethyl treatment, and the latest was at 73 days. In TELECAST, the earliest sustained improvement in BM frequency was observed at four days of telotristat ethyl treatment, and the latest was at 68 days. In TELESTAR, hazard ratios for sustained reduction of at least 30% in BM frequency were 2.3 (95% CI, 1.3–4.1) and 2.2 (95% CI, 1.3–3.9) for telotristat ethyl 250 mg tid and 500 mg tid, respectively. In TELECAST, the hazard ratios for sustained reduction of at least 30% in BM frequency were 3.9 (95% CI, 1.6–11.0) and 4.2 (95% CI, 1.7–11.7) for telotristat ethyl 250 mg tid and 500 mg tid, respectively (*p* ≤ 0.003, for each comparison versus placebo).

**Fig. 2:**
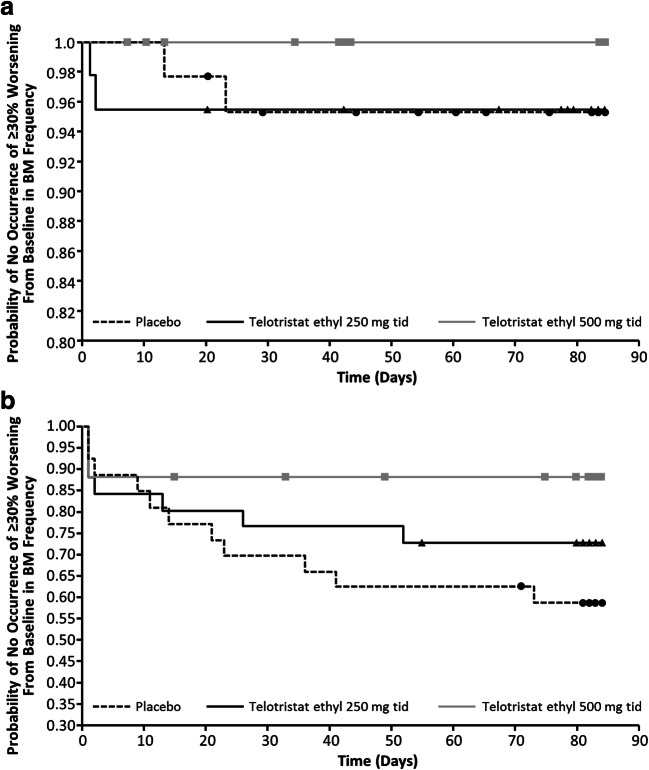
Kaplan–Meier curves for worsening in BM frequency. Probability with time that a patient would not have a ≥ 30% worsening in BM frequency for patients participating in the TELESTAR (**a**) and TELECAST (**b**) studies. The time to ≥ 30% worsening from baseline was defined as the time from the first DBT dose date to the first day of a consecutive 14-day period with BM frequency at least 30% above the individual baseline BM frequency. Patients having no ≥ 30% worsening were censored on the last day of the last week a valid assessment of the endpoint can be made. Shapes indicate censored values. Abbreviations: BM, bowel movement; DBT, double-blind treatment; tid, three times per day

Time to worsening of BM frequency was also measured for both trials (Table 3). In TELESTAR, there were very few patients with sustained worsening: 2, 2, and 0 patients with sustained worsening on placebo, telotristat ethyl 250 mg tid, and telotristat ethyl 500 mg tid, respectively, with no statistically significant difference between telotristat ethyl and placebo for either treatment arm (Fig. 2a).

**Table 3:** Number of days to first occurrence of ≥ 30% worsening from baseline in BM frequency during the DBT period

	TELESTAR			TELECAST		
Statistic	Placebo (*n* = 45)	Telotristat ethyl 250 mg (*n* = 45)	Telotristat ethyl 500 mg (*n* = 45)	Placebo (*n* = 26)	Telotristat ethyl 250 mg (*n* = 25)	Telotristat ethyl 500 mg (*n* = 25)
Patient with an event, *n*	2	2	0	11	7	3
Censored, *n*	43	43	45	15	18	22
25th percentile (95% CL)^a^, days	NR	NR	NR	21.0 (NC)	52.0 (NC)	NR
Median percentile (95% CL)^a^, days	NR	NR	NR	NR	NR	NR
75th percentile (95% CL)^a^, days	NR	NR	NR	NR	NR	NR
HR (95%), telotristat ethyl vs placebo^b^	–	1.0 (0.1, 8.4)	0.0 (NC)	–	0.5 (0.2, 1.4)	0.3 (0.1, 0.8)
*p* value^c^	–	> 0.99	0.18	–	0.18	0.021

Abbreviations: *BM*, bowel movement; *CL*, confidence limits; *DBT*, double-blind treatment; *HR*, hazard ratio; *NC*, not calculated; *NR*, not reached; *u5-HIAA*, urinary 5-hydroxyindoleacetic acid

^a^Median, percentiles, and 95% CL are from Kaplan–Meier estimates.

^b^HR and 95% CL are from Cox proportional hazards regression model, which includes treatment group and u5-HIAA stratification at randomization as fixed effects

^c^p -value is from log-rank test stratified by u5-HIAA at randomization.

In TELECAST, sustained worsening of BM frequency was more common overall, though fewer patients using telotristat ethyl experienced worsening compared with placebo (SSA alone). During the DBT period, 7 (28%) patients on telotristat ethyl 250 mg tid, 3 (12%) patients on telotristat ethyl 500 mg tid, and 11 (42%) patients on placebo had sustained worsening of BM frequency. There was a statistically significant difference between telotristat ethyl 500 mg tid and placebo (*p* = 0.021). Additionally, in the TELECAST trial, all patients were on concomitant SSA therapy, with the exception of three patients on telotristat ethyl 250 mg tid and five patients on telotristat ethyl 500 mg tid [9].

No median for time to sustained worsening of BM frequency was reached on any treatment arm in TELECAST, although 25% of patients on placebo experienced sustained worsening by day 21 compared with day 52 for telotristat ethyl 250 mg, suggesting a delay in this clinical parameter. Of note, the time to sustained worsening was further delayed on the 500 mg dose of telotristat ethyl (no percentile reached) and is statistically significant (*p* = 0.021, HR = 0.3 [95% CI, 0.06–0.8]) (Fig. 2b).

**Fig. 3:**
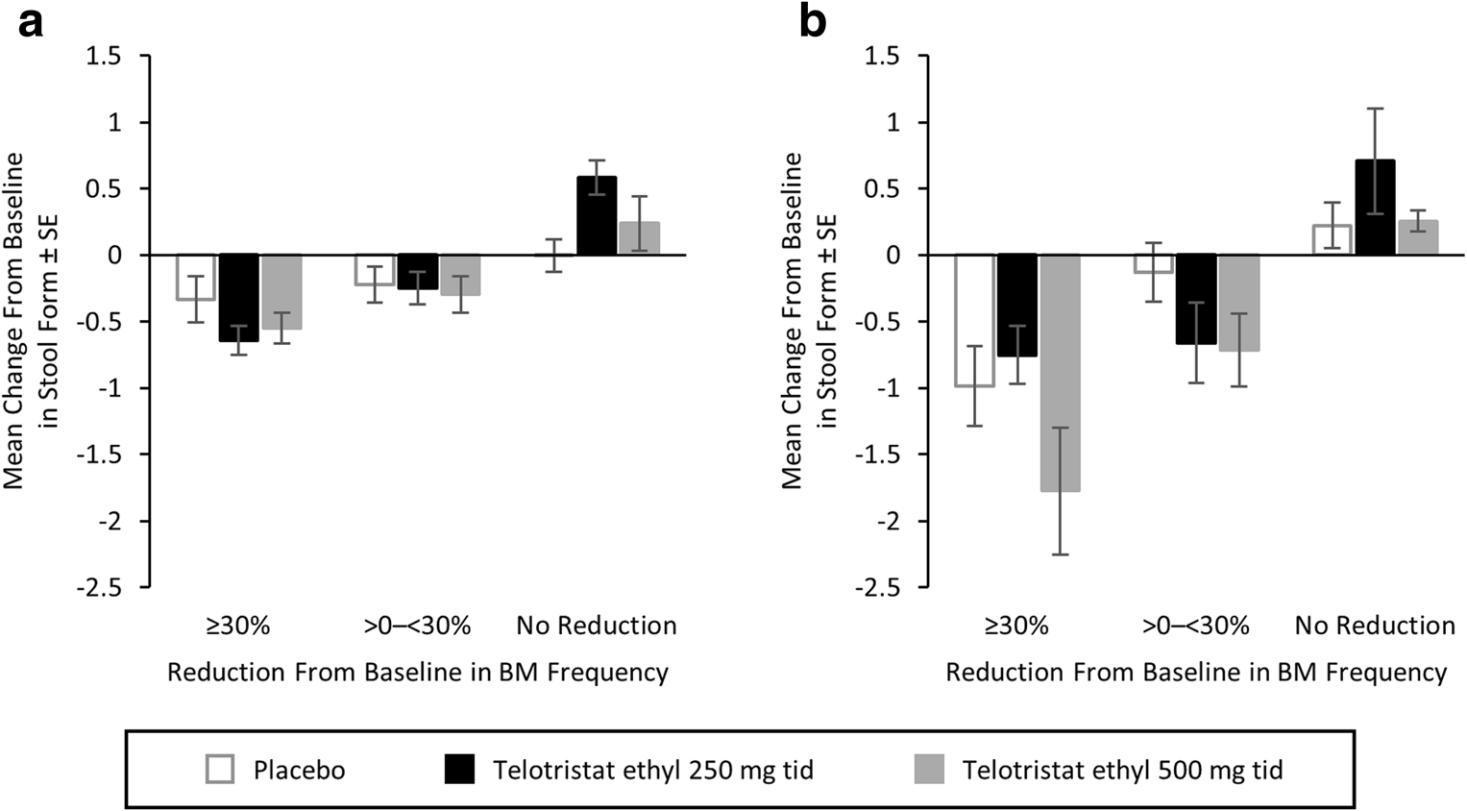
Mean change from baseline in stool form at six weeks for patients who experienced ≥ 30% reduction, 0–30% reduction, or no reduction in baseline BM frequency in the TELESTAR (**a**) and TELECAST (**b**) studies**.** The data from six weeks is presented, as both trials reached the median time to sustained improvement in BM frequency by this point. Abbreviations: BM, bowel movement; SE, standard error; tid, three times per day

**Fig. 4:**
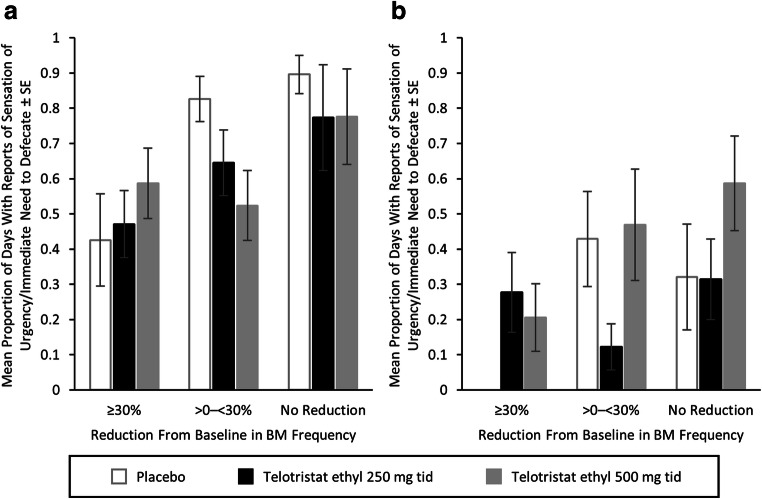
Proportion of days with reports of urgency/immediate need to defecate at 6 weeks for patients who experienced ≥ 30% reduction, 0–30% reduction, or no reduction in baseline BM frequency in the TELESTAR (**a**) and TELECAST (**b**) studies. The data from six weeks is presented, as both trials reached the median time to sustained improvement in BM frequency by this point. Abbreviations: BM, bowel movement; SE, standard error; tid, three times per day

Stool form was improved in both studies, with the greatest changes observed in the telotristat ethyl 500 mg tid group [8, 9]. Additionally, in TELESTAR, the arithmetic mean change in the proportion of days with urgency to defecate was − 0.09 (*p* = 0.35, descriptive) and − 0.15 (*p* = 0.006, descriptive) for telotristat ethyl 250 mg tid and telotristat ethyl 500 mg tid, respectively [8]. As both trials reached the median time to sustained improvement in BM frequency by six weeks, data on the mean change from baseline in stool form for patients who experienced ≥ 30% reduction, 0–30% reduction, or no reduction in BM frequency from the TELESTAR and TELECAST studies at six weeks are provided in Fig. 3a and b, respectively. In all groups, reductions in BM frequency (when they occurred) were associated with improvements in stool form. Data for the proportion of days with reports of urgency to defecate according to the patients who experienced ≥ 30% reduction, 0–30% reduction, or no reduction in BM frequency in the TELESTAR and TELECAST studies are provided in Fig. 4a and b, respectively.

## Discussion

Telotristat ethyl is an oral tryptophan hydroxylase inhibitor recently approved in the United States and Europe for the treatment of carcinoid syndrome diarrhea in combination with SSAs, on the basis of efficacy seen in two clinical trials, TELESTAR and TELECAST. Here, the time to sustained BM frequency reduction or worsening with two dose levels of telotristat ethyl was analyzed versus placebo, with almost all patients taking SSA therapy.

For both trials, median time to sustained BM frequency reduction with telotristat ethyl occurred within 19–39 days, with a range of response time of 3–73 days. In the TELESTAR study, ~ 55% of patients on telotristat ethyl showed a significant reduction in BM frequency by four weeks, 65% by eight weeks, and 75% by 12 weeks. Comparatively, BM reductions on placebo were 28%, 50%, and 55%, respectively. Given the possibility for late responses to telotristat ethyl, at least eight weeks of therapy may be needed to fully evaluate efficacy.

Sustained worsening in BM frequency was not observed in the TELESTAR study. However, in the TELECAST study, telotristat ethyl was associated with a lower incidence of worsening of BM frequency than placebo, suggesting that some patients may benefit even without a large reduction in BM frequency from baseline. This may be worth considering for patients with carcinoid syndrome and < 4 BMs per day.

Other clinical parameters appeared to improve alongside sustained improvement in BM frequency in both trials. Improvements in stool form were observed in both trials, and a reduced proportion of days with an urgency to defecate was observed in TELESTAR [4, 5]. These outcomes were more marked in the telotristat ethyl 500 mg tid dose level arms, with nonsignificant numerical decreases observed in the telotristat ethyl 250 mg tid arms [4, 5]. In TELESTAR, the 500-mg tid dose level was strongly correlated with reduced u5-HIAA levels, reduced average BM frequency, and reduced urgency to defecate [4]. Accordingly, higher baseline BM frequency may be a factor for the improvements observed at this higher dose level, as the TELESTAR study required ≥ 4 BMs per day as an inclusion criterion [4]. Of note, patients have reported urgency to defecate as one of the most important carcinoid syndrome symptoms to treat, and that carcinoid syndrome symptoms have significant impacts on their lives [18].

The reasons for the wide variability in time to onset of sustained improvement in BM frequency and the substantial numbers of patients who had responses while on placebo are unclear. The difference may relate to the high degree of variability in the systemic absorption between injections of long-acting SSAs. At a highly experienced medical center, only about half the administered SSA injections were successfully targeted to the intramuscular site, resulting in a 3-fold increase in carcinoid flushing over the following cycle [19]. Variability in the absorption of SSAs in background therapy is an important reason why efficacy is best evaluated over the course of multiple long-acting injections. In both trials, a three- to four-week baseline assessment of BM frequency was used based on the usual dosing frequency for SSAs and the DBT period of 12 weeks in both trials encompassed three or four injections of SSAs. The length of time a patient was on SSA therapy may also play a role, as it is known that patients may become refractory to SSAs over time [1, 11, 20].

Diet may be another reason for variability in onset of effect and is an important consideration when taking telotristat ethyl for carcinoid syndrome. Dietary composition was not specifically evaluated during these studies. It is recommended that the medication be taken with meals. Administration of telotristat ethyl with a high-fat meal resulted in improved systemic exposure to telotristat ethyl, with a *C*_max_ (peak plasma concentration) and AUC_0–inf_ (area under the concentration-time curve from 0 to infinity) 112% and 264% higher, respectively, compared with the fasted state [5]. A lack of response during initial therapy with telotristat ethyl could potentially be addressed by ensuring that telotristat ethyl is taken with proper meals, and that pancreatic enzymes are used if fat malabsorption is suspected [20]. This point may be especially relevant to patients who limit fat intake because of their carcinoid syndrome.

Patients with diarrhea from causes other than carcinoid syndrome were excluded from these studies. However, diarrhea in these patients is often multifactorial. Factors contributing to diarrhea might include carcinoid-related bioactive compounds (serotonin and others), pancreatic enzyme deficiency, bile acid loss after intestinal surgery, shortened bowel, and bacterial overgrowth. Although it is unclear whether there would be additional patients responding to telotristat ethyl after 12 weeks of therapy, there does seem to be a plateau of onset of effects by that time (approximately 75% of patients in the TELESTAR study had a sustained response). Some patients are likely to have diarrhea predominantly related to factors other than serotonin produced by NETs, which is the only cause of diarrhea expected to be sensitive to telotristat ethyl. This may account for some of the approximately 30% of patients who did not seem to respond to telotristat ethyl.

The reliability of the BM frequency measurements was a strength of these studies. Electronic diaries provided time-stamped daily assessments, leading to approximately 10,000 measures of BM frequency in the TELESTAR study alone. This allowed an ample basis for careful review of time patterns. A key limitation of the studies was the inability to comprehensively examine all the different variables that can affect BM frequency in clinical trials. For instance, neither diet nor individual doses of over-the-counter diarrhea treatments were tracked on a daily basis. Short-acting octreotide use was examined, and its use was reduced on telotristat ethyl compared with placebo (SSA alone) in the TELESTAR study [8]. More information on these and other variables could potentially help maximize efficacy.

In conclusion, sustained reductions of at least 30% in BM frequency on telotristat ethyl were observed within 3 to 6 weeks in half of the patients in these phase III trials with a range of onset between three and 73 days. Results of the trials suggest that some patients on telotristat ethyl may also have benefited by avoiding a worsening in BM frequency. Additionally, the telotristat ethyl dose level correlated with improvements in stool form and urgency. These parameters were generally associated with BM frequency and may help in the evaluation of telotristat ethyl efficacy. Understanding the time course of telotristat ethyl effects may help ensure that patients and their care providers have appropriate expectations and guide the initial assessment of the drug in patients with carcinoid syndrome diarrhea.
